# Single-cell RNA-seq reveals a link of ovule abortion and sugar transport in *Camellia oleifera*

**DOI:** 10.3389/fpls.2024.1274013

**Published:** 2024-02-02

**Authors:** Songzi Zhao, Jun Rong

**Affiliations:** ^1^ Jiangxi Province Key Laboratory of Camellia Germplasm Conservation and Utilization, Jiangxi Academy of Forestry, Nanchang, China; ^2^ Jiangxi Province Key Laboratory of Watershed Ecosystem Change and Biodiversity, Center for Watershed Ecology, Institute of Life Science and School of Life Sciences, Nanchang University, Nanchang, China

**Keywords:** *Camellia oleifera*, single-cell RNA sequencing, ovary, ovule abortion, selective abortion, sugar transport

## Abstract

*Camellia oleifera* is the most important woody oil crop in China. Seed number per fruit is an important yield trait in *C. oleifera*. Ovule abortion is generally observed in *C. oleifera* and significantly decreases the seed number per fruit. However, the mechanisms of ovule abortion remain poorly understood at present. Single-cell RNA sequencing (scRNA-seq) was performed using mature ovaries of two *C. oleifera* varieties with different ovule abortion rates (OARs). In total, 20,526 high-quality cells were obtained, and 18 putative cell clusters were identified. Six cell types including female gametophyte, protoxylem, protophloem, procambium, epidermis, and parenchyma cells were identified from three main tissue types of ovule, placenta, and pericarp inner layer. A comparative analysis on scRNA-seq data between high- and low-OAR varieties demonstrated that the overall expression of *CoSWEET* and *CoCWINV* in procambium cells, and *CoSTP* in the integument was significantly upregulated in the low-OAR variety. Both the infertile ovule before pollination and the abortion ovule producing after compatible pollination might be attributed to selective abortion caused by low sugar levels in the apoplast around procambium cells and a low capability of hexose uptake in the integument. Here, the first single-cell transcriptional landscape is reported in woody crop ovaries. Our investigation demonstrates that ovule abortion may be related to sugar transport in placenta and ovules and sheds light on further deciphering the mechanism of regulating sugar transport and the improvement of seed yield in *C. oleifera*.

## 1 Introduction

*Camellia oleifera* is the most important woody oil crop in China. Seed oil of *C. oleifera* is rich in oleic acid (>80%), known as “oriental olive oil”, and contains many biological active components such as phytosterols, squalene, vitamin E, polyphenols, and saponin. With the rapid economic development and the increase in needs of healthy vegetable oil over the past decades, *C. oleifera* has become one of the most important edible oil plants in China at present. However, the production of camellia oil is still very low, which has become a bottleneck of the industry development. For instance, in, 2019, there was approximately 198 kt camellia oil produced in Jiangxi province of China, one of the major provinces for camellia oil production, but the average yield was only 190 kg hm^−2^. On the other hand, as a perennial woody crop, the traditional breeding of *C. oleifera* is inefficient and time consuming. Therefore, determining the molecular bases of important yield traits may facilitate the molecular breeding of *C. oleifera* with high yield.

Seed number per fruit is an important yield trait in *C. oleifera*. Ovule abortion is generally observed in *C. oleifera* and significantly decreases the seed number per fruit. The mechanisms of ovule abortion remain poorly understood in *C. oleifera* nowadays. There are approximately 15 ovules in a fruit with three carpels, which is a typical carpel number in *C. oleifera*. Seed number per fruit was approximately 1.58–9.14 in 118 C*. oleifera* germplasm resources (Lin et al., 2022), and 102 of them had less than five seeds per fruit. Self-incompatibility may result in ovule abortion to some extent (Liao, 2013; Liao et al., 2014; Gao, 2017; He et al., 2020; Zhou et al., 2020; Li et al., 2023). Moreover, it was found that the infertile ovules that presented when embryonic sac matured, instead of developing after pollination, were another source of abortion ovules (Gao, 2017). The proportions of infertile ovules in ovaries were 13.3%–20.2%, 15.6%–33.3%, 10.2%–20.5%, and 20.8%–30.7% in *C. oleifera* varieties ‘Huashuo’, ‘Huajin’, ‘Huaxin’, and ‘Xianglin XLC15’, respectively (Gao, 2017). According to the published data of Gao (2017), we estimated that the ovule abortion rates (OARs) of ‘Huashuo’ after cross-pollination with ‘Huaxin’, ‘Huajin’, and ‘Xianglin XLC15’ were 62.2%, 60.0%, and 57.4%, respectively, which were approximately threefold higher than its proportion of infertile ovules. Approximately 40% abortion ovules were produced after compatible pollination in ‘Huashuo’. Therefore, there may be three types of abortion ovules in *C. oleifera*: 1) resulted from infertile ovules before pollination; 2) caused by self-incompatibility; and 3) produced after compatible pollination.

In *Arabidopsis*, embryo mutants with defects in female gametophyte (FG), such as *capulet1* (Grini et al., 2002), *zak ixik* (Ngo et al., 2012), *oiwa* (Martin et al., 2013), and *Athemn1-1* (Pratibha et al., 2017), showed development arrest at different stages. In the *capulet1* mutant, embryo sacs were already abnormal at the zygote stage, and approximately 29%, 41%, and 13% of embryos were arrested as zygotes, one-nucleate proembryos, and two-nucleate proembryos, respectively (Grini et al., 2002). In the *Athem1-1* mutant, approximately 12% of ovules showed arrest at the FG1, FG2, or FG4 stage of female gametophyte development, approximately 28% of the aborting seeds had embryos arrested at the globular stage, and approximately 31% reached the torpedo or cotyledon stage (Pratibha et al., 2017). The OARs of ‘Xianglin XLC15’ were 85.5% 35 days after self-pollination and 46.3% 22 days after cross-pollination and then were relatively stable until the seeds reached maturity (Liao, 2013; Liao et al., 2014). Therefore, ovule abortion in *C. oleifera* may be a continuous process lasting from the stage of embryonic sac maturity to the stage of early zygote (approximately 35 days after anthesis) in open pollination.

Gao (2017) found that infertile ovules were commonly located in the middle and lower sections of the ovary. As far as we know, ovule abortion usually happened randomly in fruits except for the abnormality of transmitting tract (Gremski et al., 2007; Crawford and Yanofsky, 2011; Di Marzo et al., 2020) and selective abortion (Guitian, 1994; Xue-Jie and Dun-Yan, 2007; Arathi, 2011). Muralla et al. (2011) suggested that embryo mutants with defects in female gametophyte development typically had a low percentage of mutant seeds, randomly distributed along the silique, combined with a high percentage of aborted ovules after analyzing 396 embryo-defective genes of *Arabidopsis*. The *hec3*, a loss-of-function mutant of AtHEC3 (AT5G09750), which regulated transmitting tract development, had a modest reduction in fertility compared with the wild type (59% wild-type seed set), and a biased seed distribution toward the apical half of the carpel because the pollen tubes that reached the base of the ovary were fewer in number than that in the wild type (Gremski et al., 2007). Selective abortion is a survival strategy adopted by many species that sacrifice some seeds/fruits to allow the remaining ones to survive according to pollen source, order of pollination, location of fruits on plant, number of developing seeds, or some combinations of these. The primary factor causing selective abortion is resource limitation (Stephenson, 1981; Bawa and Webb, 1984; Medrano et al., 2000). In maize, when apical and basal kernels were synchronously pollinated, the basal kernels set and matured, but the apical kernels were aborted at an early stage owing to a low level of local assimilates (Shen et al., 2018). Delaying pollination to the basal ovaries reduced activity of cell wall acid invertase (CWINV) and sugar levels, which allowed the apical kernels to set and grow normally (Shen et al., 2018). The distribution of vascular in the ovary was also an important factor leading to selective abortion (Horovitz et al., 1976).

We speculated that the abortion ovules resulting from infertile ovules before pollination in *C. oleifera* might be attributed to selective abortion caused by resource limitation. Single-cell RNA sequencing (scRNA-seq) has provided a powerful tool to analyze gene expression in thousands of individual cells from a heterogeneous tissue. In plants such as *Arabidopsis* (Denyer et al., 2019), rice (Liu et al., 2021a), *Populus* (Li et al., 2021), woody strawberry (Bai et al., 2022), and tea tree (Wang et al., 2022a), scRNA sequencing efforts have been reported, providing clues to identify cell types in other plant species. Here, we constructed the first single-cell level atlas of *C. oleifera* mature ovaries. A group of genes related to ovule abortion, which participated in sugar transport in placenta or ovules, were identified by comprehensively comparing gene expressions between high- and low-OAR varieties in *C. oleifera*. Our study may help understand the molecular mechanisms of ovule abortion in *C. oleifera* and facilitate the molecular breeding of high-yield varieties with reduced ovule abortion.

## 2 Materials and methods

### 2.1 Plant growth and sample collection

*Camellia oleifera* varieties ‘GW’ (CoGW) and ‘XJ’ (CoXJ) were planted in the *Camellia* Gene Bank at Jiangxi Academy of Forestry (28°41′N, 115°48′E), Nanchang, Jiangxi Province, China. The ovule abortion rate (OAR) was analyzed using mature fruits. Mature ovaries were collected when the plants began flowering in November. The epicarp was quickly removed from all collected ovaries before further study. Each sample contained 12 mature ovaries. To isolate high-quality protoplasts, we cut out the epicarp of the ovaries because the woody epicarp and its attached trichomes resulted in lower viability of the protoplasts and more cell debris.

### 2.2 Tissue digestion and protoplast isolation

Ovary tissues were cut into pieces and placed in RNase-free enzyme solution (1.5% [w/v] cellulose R10, 0.5% [w/v] pectinase, 0.5 M mannitol, 20 mM KCl, 10 mM MES [pH 5.7], 10 mM CaCl_2_, and 0.1% [w/v] bovine serum albumin). The tissues were enzymolyzed at 75 rpm for 4 h at 30°C in the dark. The digestion mixture was filtered through a 40-μm filter. Protoplasts were centrifuged at 150 g for 5 min once and 150 g for 3 min twice, and washed with WB solution (0.5 M mannitol, 0.1% [w/v] bovine serum albumin). The activity of single-cell suspensions was detected by 0.4% trypan blue staining, and protoplasts with >90% activity were selected for further analysis. The density of the protoplasts was determined with a hemocytometer and adjusted to 1,000–2,000 cells/μL.

### 2.3 scRNA-seq library construction, sequencing, and raw data quality control

Cellular suspensions were loaded on a 10X Genomics GemCode single-cell instrument that generated single-cell Gel Bead-In-EMlusion (GEMs). Libraries were generated from the cDNAs with Chromium Next GEM Single Cell 3′ Reagent Kits v3.1 and were sequenced on the Illumina sequencing platform (PE150) by Genedenovo Biotechnology Co., Ltd (Guangzhou, China).

The raw data in FASTQ format were processed to obtain clean reads. *Camellia oleifera* var. ‘Nanyongensis’ (CON) genome (Lin et al., 2022) was used as the reference genome. The output of Cell Ranger software (version 3.1.0) was loaded into Seurat (version 3.1.1) (Butler et al., 2018), which was used for dimensional reduction, clustering, and analysis of scRNA-seq data. Only reads that were uniquely mapped were used for UMI (Unique Molecular Identifier) counting. Cells with unusually high number of UMIs (≥22,000) were filtered out. We also excluded cells with<360 or >4,000 genes detected. The cells with UMI numbers >22,000 were likely two or more cells in one drop. The cells with<360 genes were considered low quality, and the cells with >4,000 genes were likely to be two or more cells in one drop. Additionally, doublet GEMs were also filtered out. It was achieved using the tool DoubletFinder (v2.0.3) by the generation of artificial doublets, using the PC (principal component) distance to find each cell’s proportion of artificial k nearest neighbors (pANN) and ranking them according to the expected number of doublets (McGinnis et al., 2019).

### 2.4 Cell clustering

After removing unwanted cells from the dataset, we employed a global-scaling normalization method “LogNormalize” that normalized the gene expression measurements for each cell by the total expression, multiplied this by a scale factor (10,000 by default), and log-transformed the results. The formula was showed as follows:


(1) $$\text{A}\;\text{gene}\;\text{expression}\;\text{level}\;=\;\text{log}\;\left(1\;+\;10000\;\times \;\text{U}\text{M}{\text{I}}_{\text{A}}/\text{U}\text{M}{\text{I}}_{\text{Total}}\right)$$

In Equation 1, UMI represents the Unique Molecular Identifier, UMI_A_ is the Unique Molecular Identifier of gene A, and UMI_Total_ is the Unique Molecular Identifier of total genes.

Then, we implemented a resampling test inspired by the jackStraw procedure. We randomly permuted a subset of the data (1% by default) and rerun PCA (principal component analysis), constructing a “null distribution” of gene scores, and we repeated this procedure. We identified “significant” PCs as those who had a strong enrichment of low p-value genes for downstream clustering and dimensional reduction (Chung and Storey, 2015). Distances between the cells were calculated based on the identified PCs. Briefly, Seurat embed cells in a shared-nearest neighbor (SNN) graph, with edges drawn between cells via similar gene expression patterns. To partition this graph into highly interconnected quasi-cliques or communities, we first constructed the SNN graph based on the Euclidean distance in PCA space and refined the edge weights between any two cells based on the shared overlap in their local neighborhoods (Jaccard distance). We then clustered cells using the Louvain (Rotta and Noack, 2011) method to maximize modularity. For visualization of clusters, t-distributed Stochastic Neighbor Embedding (t-SNE) (van der Maaten and Hinton, 2008) was generated using the same PCs.

### 2.5 Differentially expressed gene analysis

For DE genes in cell clusters, expression value of each gene in a given cluster was compared against the rest of cells using Wilcoxon rank sum test (Camp et al., 2017). For DE genes between CoXJ and CoGW, expression value of each gene in a given cluster of CoXJ was compared against the counterparts of CoGW. Significantly upregulated genes were identified using a number of criteria. First, genes had to be at least 1.28-fold overexpressed in the target cluster. Second, genes had to be expressed in more than 25% of the cells belonging to the target cluster. Third, p-value was<0.01.

### 2.6 RNA-seq expression analysis

In our study, transcriptome data from various tissues of both *C. oleifera* and *C. chekiangoleosa* were obtained in the public database NCBI or Genome Sequence Archive (https://ngdc.cncb.ac.cn/gsa/) (Chen et al., 2021) with three biological replicates. The RNA-seq reads, including SRR8275897/902 (pistil), SRR10121540-46/48-57/59 (*C. chekiangoleosa* flower bud, seed coat, seed kernel, epicarp, mesocarp, endocarp), SRR13493686-88/90-98 (seed), SRR17155283-85/90-92 (cotyledon), SRR21160439-41 (pericarp), and SRR23501938-40 (root) were obtained from NCBI, while CRR180020-25 (leaf), CRR400359-67 (anther), and CRR274890-892/896-898/905-907 (flower bud without sepal and pedicel) were from Genome Sequence Archive. We used some RNA-seq data from *C. chekiangoleosa* because of no data on seed coat in *C. oleifera*. The Bowtie 2 (Langmead and Salzberg, 2012) was used to map the reads to CoSWEET transcript sequences (**Supplementary Table S1**) with default parameters. The expression levels of the genes were calculated and compared using the FPKM method (fragments per kb per million fragments).

### 2.7 Expression analysis of genes from seven families related to sugar transport

The scRNA-seq reads were map to the transcript sequences (**Supplementary Table S1**) of genes from seven families (SWEET, ACINV, ANInv, SUS, STP, PLT, and SUC) related to sugar transport with default parameters using Bowtie 2. The formula for calculating gene expression values was shown as follows:


(2) $$\text{A}\;\text{gene}\;\text{expression}\;\text{value}\;=\;10000\;\times \;\text{N}\text{M}{\text{R}}_{\text{A}}/\text{U}\text{M}{\text{I}}_{\text{Total}}$$

In Equation 2, NMR_A_ was the number of matched reads of gene A. The significance of the genes related to the sugar transport between CoXJ and CoGW was determined at p ≤ 0.05 and p ≤ 0.01 by analysis of variance (ANOVA).

The transcript sequences of genes from seven families were used as the reference genome because the sequences of some genes in those families could not be found in *C. oleifera* var. ‘Nanyongensis’ (CON) genome (Lin et al., 2022) owing to its imperfection.

## 3 Results

### 3.1 *Camellia oleifera* variety ‘XJ’ had a low ovule abortion rate

*Camellia oleifera* variety ‘XJ’ (CoXJ) is a superior low-OAR variety (**Supplementary Figure S1A**) discovered from the germplasm resources in the *Camellia* Gene Bank at Jiangxi Academy of Forestry, Nanchang, Jiangxi Province, China. Seed number per fruit was approximately 11.62–14.00 in CoXJ (**Supplementary Table S2**) whose carpel number was approximately 3.00, and higher than that of approximately 2.98–3.38 in *C. oleifera* variety ‘GW’ (CoGW) whose carpel number was approximately 3.16 (Zhao and Xing, 2023). Compared with the reported 118 C*. oleifera* germplasm resources (Lin et al., 2022), CoXJ was the highest in seed number per fruit. Ovule number per fruit and ovule number per locule were approximately 16.34 (**Supplementary Table S3**) and 5.44, respectively, in CoXJ, while ovule number per locule were approximately 5.04 in CoGW (Zhao and Xing, 2023). CoXJ had an OAR of 28.9% on average in open-pollination (**Supplementary Table S3**). Approximately 3% fruits bearing upper outside the canopy had no abortion ovule in CoXJ. CoGW is a high-OAR variety (**Supplementary Figure S1B**) with an OAR of 78.2% on average (Zhao and Xing, 2023). The results of cross-experiment showed that maternal inheritance contributed substantially to the low-OAR trait of CoXJ (**Supplementary Table S2**).

### 3.2 Summary of mature ovary scRNA-seq in *C. oleifera*

**Figure 1:**
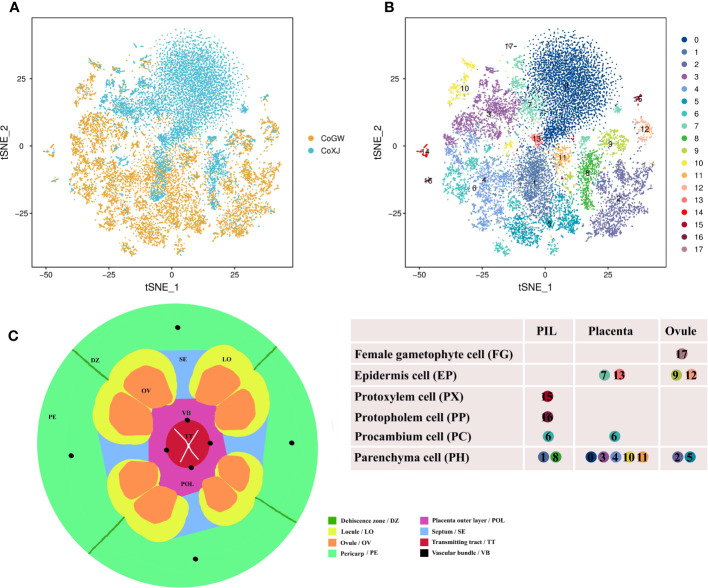
Overviews of the cell atlas of *Camellia oleifera* mature ovaries. **(A)** Visualization of cell of CoGW and CoXJ using tSNE. Dots, individual cells; n = 20,526 cells; color, varieties. CoGW, *C. oleifera* variety ‘GW’; CoXJ, *C*. *oleifera* variety ‘XJ’. **(B)** Visualization of 18 cell clusters using tSNE. Dots, individual cells; n = 20,526 cells; color, cell clusters. PIL, pericarp inner layer (including mesocarp and endocarp); placenta, including septum. **(C)** Schematic cross-section showing the different tissues of a mature ovary.

We performed scRNA-seq using the 10× Genomics Chromium platform (Zheng et al., 2017) to study ovary development in *C. oleifera*. Protoplasts were prepared from mature (the day of anthesis) ovaries of both CoGW and CoXJ. In total, 22,713 cells (12,713 in CoGW; 10,000 in CoXJ) were captured for library construction and paired-end sequencing (PE150). After filtration removal of the cells with gene numbers<360 and over >4,000 (**Supplementary Figures S2A, B**) and the cells with more than 22,000 UMIs (**Supplementary Figures S2C, D**), a total of 20,526 high-quality cells (11,454 in CoGW; 9,072 in CoXJ) were retained for further analysis. In total, 779,251,694 sequencing reads (388,422,932 in CoGW; 390,828,762 in CoXJ) were obtained, with 95.3% valid barcodes. Of the sequencing reads, 77.0% could be mapped to the *C. oleifera* var. ‘Nanyongensis’ (CON) genome (Lin et al., 2022). A total of 34,031 genes were identified in these 20,526 cells, with median gene number of 1,294 per cell, and the median UMI number per cell was 2,427 (**Supplementary Table S4**). We conducted unsupervised clustering analysis of the 20,526 cells with the canonical correlation analysis function of Seurat (Butler et al., 2018), which yielded transcriptionally distinct 18 clusters (**Figures 1A, B**). The cell numbers distributed in each cluster ranged from 31 to 5,404 (**Supplementary Table S5**).

### 3.3 Cell types of 18 clusters

To provide clues for defining cell types of the 18 clusters, the structure of a mature ovary (**Figure 1C**) was drawn based on the results of cross-sectional observation (Cao, 1965; Liao, 2013; Liao et al., 2014; Gao et al., 2015; Gao, 2017; Gao et al., 2019). Upregulated differentially expressed (DE) genes were identified in each cluster (**Supplementary Table S6**) using a fold change FC > 1.28 and a p-value< 0.01 compared with the other 17 clusters, and more than 25% expression of the cells belonging to the target cluster. The potential functions and pathways of the genes were determined according to Kyoto Encyclopedia of Genes and Genomes (KEGG) and Gene Ontology (GO) analyses.

**Figure 2:**
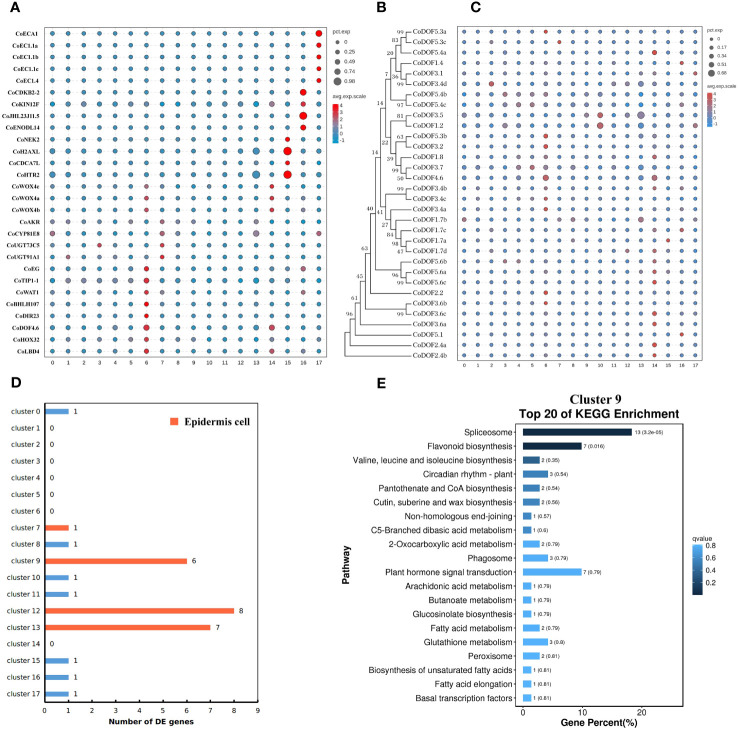
Cell type definition of 18 clusters from *Camellia oleifera* mature ovaries. **(A)** Cell type definition of clusters 6, 7, 14–17 using cell-specific genes from other plants. Clusters 6 and 14, procambium cells; cluster 7, epidermis cells; cluster 15, protoxylem cells; cluster 16, protophloem cells; cluster 17, female gametophyte cells. **(B, C)** Identification of cluster 14 as a fruit-special procambium cell different from cluster 6. **(B)** Neighbor-joining phylogenetic tree of CoDOF proteins was created using MEGA7. **(C)** A total of 11 *CoDOF* genes were highly expressed in cluster 14. **(D)** The number of DE genes involved in cutin and cuticular wax biosynthesis in 18 clusters. Clusters 9, 12, and 13 were defined as epidermis cells. **(E)** KEGG enrichment analysis of the DE genes in cluster 9. KEGG pathway “cutin, suberine and wax biosynthesis” (ko00073) was enriched in cluster 9.

At first, cluster 17 was defined as a female gametophyte cell. We checked the cell-specifically expressed genes among the DE genes (**Supplementary Table S6**) that were significantly and specifically upregulated in the cluster and found a homologue (*CoECA1*) of synergid cell (SE) marker gene *AT3G30247* (Song et al., 2020), which encoded a ECA1 gametogenesis-related family protein, and four homologues (*CoEC1.4*, *CoEC1.1a*, *CoEC1.1b*, and *CoEC1.1c*) of egg cell (EC) marker gene *AT4G39340* and *AT1G76750* (Song et al., 2020), which encoded small cysteine-rich proteins secreted by EC (**Figure 2A**; **Supplementary Table S7**). Cluster analysis of all 31 cells in cell cluster 17 showed that there were two ECs and seven SEs using top 1,000 variable genes in the cell cluster (**Supplementary Figure S3**).

All genes whose expression has previously been assigned to early stages of vascular development in the leaf have also been reported to be expressed in root vascular cells (Gardiner et al., 2010). Therefore, we speculated that vascular bundle (VB) cell markers from leaves of tea plant were suitable to *C. oleifera* mature ovaries, and defined cluster 6 as a procambium (PC) cell, cluster 15 as a protoxylem (PX) cell, and cluster 16 as a protophloem (PP) cell using a group of cluster-specific genes from tea plant (**Figure 2A**; **Supplementary Table S7**) (Wang et al., 2022a). At the same time, we found that a homologue (*CoDOF4.6*) of a PC marker gene *CSS0039005* (Wang et al., 2022a) (a homologue of *AT4G24060*) was also a DE gene of cluster 14, and other transcription factors needed for vascular development such as CoWOX4a, CoWOX4b, CoWOX4c (a homologue of AT1G46480) (Hirakawa et al., 2010), CoHOX32 (a homologue of AT2G34710) (Bertolotti et al., 2021), and CoLBD4 (a homologue of AT1G31320) (Smit et al., 2020) were upregulated in cluster 14. DNA-BINDING WITH ONE ZINC FINGER (DOF) gene family encodes plant-specific transcription factors with 36 members in *Arabidopsis*, nine of which display strong expression in root vascular cells (Gardiner et al., 2010). Therefore, we checked the expression of 32 DOF genes in *C. oleifera* and found that seven DOF genes were highly expressed in cluster 6, while 11 DOF genes were preferentially expressed in cluster 14 (**Figures 2B, C**; **Supplementary Table S8**), which was defined as a fruit-special PC cell.

Because the remaining clusters were epidermis (EP) cells, which were known to express wax/cutin biosynthetic genes for synthesizing cuticular wax and cutin (Suh et al., 2005), or parenchyma (PH) cells, some of which had low levels of cuticle biosynthetic genes and relatively high levels of photosynthetic genes, we employed another strategy that had been used in corolla of the wild tobacco (Kang et al., 2022) to annotate remaining cell clusters from *C. oleifera*. The DE genes involved in cutin and cuticular wax biosynthesis were enriched in clusters 9, 12, and 13, whose DE gene numbers were 6, 8, and 7, respectively (**Figure 2D**; **Supplementary Table S9**). GO enrichment analysis showed that several cellular components including “photosystem” (GO:0009521), “photosynthetic membrane” (GO:0034357), “photosystem I” (GO:0009522), and “photosystem II” (GO:0009523) were enriched in cluster 10 mostly, and in clusters 1, 3, and 4 in some extend compared to other clusters (**Supplementary Figures S4A–D**). KEGG enrichment analysis showed that the pathway “cutin, suberine and wax biosynthesis“ (ko00073) was enriched in cluster 9 (**Figure 2E**), and the pathway “photosynthesis“ (ko00195) was enriched in cluster 2 (**Supplementary Figure S4E**). These results suggested that clusters 9, 12, and 13 were epidermal cells, while clusters 1, 2, 3, 4, and 10 were parenchyma cells.

Finally, we checked the expression of epidermis-special marker genes from leaves of tea plant in clusters 0, 5, 7, 8, and 11. Four genes (*CoAKR*, *CoCYP81E8*, *CoUGT73C5*, and *CoUGT91A1*), which are homologous genes of *CSS0021116*, *CSS0017885*, *CSS0031056*, and *CSS0002396* (Wang et al., 2022a), respectively, were highly expressed in cluster 7 compared to other clusters (**Figure 2A**; **Supplementary Table S7**). The transcriptome profiles of clusters 7 and 13 were highly correlated (Pearson’s correlation, r = 0.984) (**Supplementary Figure S5**). These results suggested that cluster 7 was a putative epidermal cell, while clusters 0, 5, 8, and 11 were putative parenchyma cells without photosynthesis.

### 3.4 Tissue types of 18 clusters

The identification of cell types can help us identify tissue types of 18 clusters and vice versa. There were three main tissue types: ovule (OV), placenta (including septum; PL), and pericarp inner layer (including mesocarp and endocarp; PIL). Obviously, cluster 17 was from OV according to its cell type.

**Figure 3:**
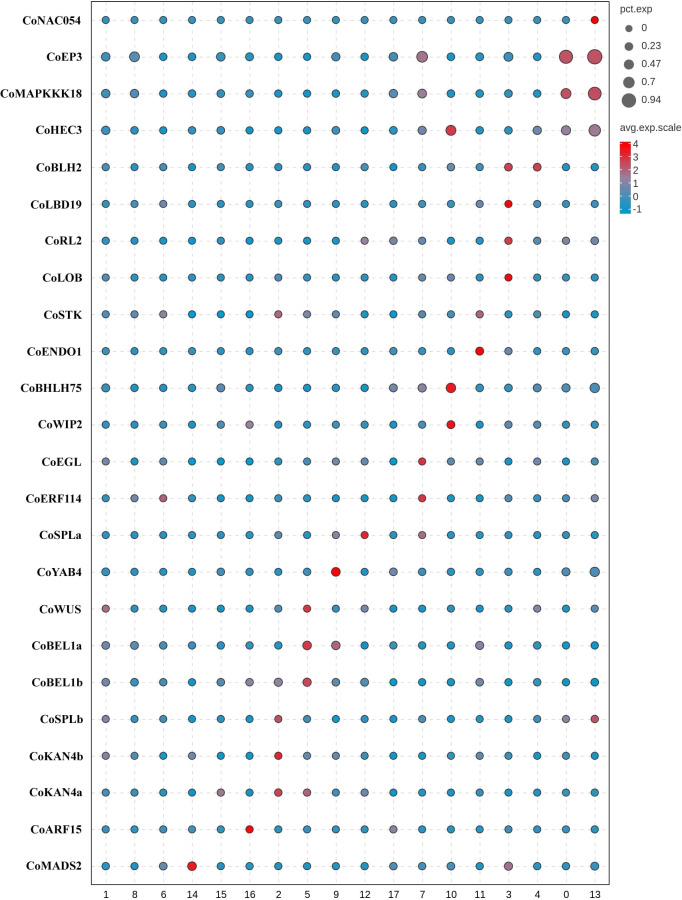
Tissue type definition of 18 clusters from *Camellia oleifera* mature ovaries using tissue-specific genes.

We employed three groups of tissue-specific genes (**Figure 3**, **Supplementary Table S10**) to identify clusters 0, 3, 4, 7, 10, 11, and 13 as placenta tissues. The first group of genes were *CoNAC054* (a homologue of *AT3G15170/AtNAC054*), *CoEP3* (a homologue of *Solyc06g053380*), and *CoMAPKKK18* (a homologue of *Solyc07g064820*), which were highly expressed in cluster 0 or 13, and *CoHEC3* (a homologue of *AtHEC3* and *Solyc11g005780*), which was a DE gene of clusters 0, 10, and 13 (**Supplementary Table S6**). *AtNAC054* was sole placenta-specific experimental marker in PCMDB (Jin et al., 2022). Both *Solyc06g053380* and *Solyc07g064820* were reported to be highly expressed in the septum of wild tomato at anthesis (Pattison et al., 2015), while *Solyc11g005780* was preferentially expressed in the placenta and the septum (Pattison et al., 2015). RNA *in situ* analysis showed that *AtHEC3* expression was first observed in the developing septum and transmitting tract during stage 8 of flower development. *AtHEC3* continued to be strongly expressed in the transmitting tract during late stage 12 (Gremski et al., 2007). The second group of genes were *CoLBD19* (a homologue of *Solyc01g091400*), *CoRL2* (a homologue of *Solyc10g052470*), *CoLOB* (a homologue of *Solyc06g071660* and *AT5G63090/AtLOB*), and *CoBLH2* (a homologue of *AT4G36870/AtBLH2*), which were highly expressed in cluster 3 or 4. *Solyc01g091400*, *Solyc10g052470*, and *Solyc06g071660* were reported to be specifically expressed in the placenta of wild tomato at anthesis (Pattison et al., 2015). *AtBLH2*, a member of the BELL family, was expressed in the transmitting tract (Kumar et al., 2007). *AtLOB* was expressed in a band of cells at the adaxial base of all lateral organs formed from the shoot apical meristem and at the base of lateral roots (Shuai et al., 2002). The cells expressing *CoLOB* might be at the base of lateral funiculus in the placenta. Therefore, we believed that clusters 3 and 4 might be from the septum and placenta outer layer (POL), the outer layer of axile placenta. The third group of genes were *CoHEC3*, *CoBHLH75* (a homologue of *AT1G25330/AtBHLH75*) (Crawford and Yanofsky, 2011; Di Marzo et al., 2020), and *CoWIP2* (a homologue of *AT3G57670/AtWIP2*) (Herrera-Ubaldo et al., 2019), which were highly expressed in cluster 10; *CoSTK* (a homologue of *AT4G09960/AtSTK*) (Herrera-Ubaldo et al., 2019) and *CoENDO1* (a homologue of *AT1G11190/AtENDO1*), which were highly expressed in cluster 11; and *CoEGL* (a homologue of *AT4G09740*) and *CoERF114* (a homologue of *AT1G43160*), which were highly expressed in cluster 7. *CoHEC3*, *CoBHLH75*, *CoWIP2*, and *CoSTK* were predicted transcription factors required for the transmitting tract development according to the functions of their homologues. *AtENDO1* was a marker gene of the programmed cell death highly expressed in the transmitting tract (Tung et al., 2005; Farage-Barhom et al., 2008). Both *AT4G09740* and *AT1G43160* were transmitting tract-specific genes (Tung et al., 2005). Therefore, we believed that clusters 7, 10, and 11 might be from the transmitting tract of axile placenta.

Next, a group of genes (**Figure 3**; **Supplementary Table S10**), including *CoWUS*, *CoYAB4*, *CoSPLa*, *CoSPLb*, *CoBEL1a*, *CoBEL1b*, *CoKAN4a*, and *CoKAN4b*, which were homologues of integument-specific experimental markers (*AT2G17950*/*AtWUS*, *AT1G23420*/*AtYAB4*, *AT4G27330*/*AtSPL*, *AT5G41410*/*AtBEL1*, and *AT5G42630*/*AtKAN4*) in PCMDB (Jin et al., 2022), were utilized to determine that clusters 2, 5, 9, and 12 were OV cells. AtYAB4, mRNA was first detected in a group of approximately 15 epidermal cells on the abaxial half of each ovule primordium, prior to visible emergence of the integuments, detected only in the outer integument (OI) on the abaxial side of the ovule primordium at stage 2-II/III, and no longer present at detectable levels at anthesis using *in situ* hybridization in *Arabidopsis* (Villanueva et al., 1999). However, the fusion gene (P-SlINO::*SlINO : GFP*) of *SlINO* (*AtYAB4* ortholog) and green fluorescent protein (GFP) was first visible after the emergence of the integument, remained high expression in the outer layer of the integument at anthesis, and continued to be visible through the onset of fruit development (Skinner et al., 2016). The expression of *YAB4* in the outermost cell layer of the outer integument was conserved in early diverging bitegmic angiosperms such as *Cabomba caroliniana* (Nymphaeales) and *Annona squamosa* (Magnoliales) (Lora et al., 2011; Yamada et al., 2011). Thus, we defined cluster 9 as an OI epidermal cell according to high expression of *CoYAB4* in the cluster and defined cluster 5 as an OI parenchyma cell owing to high expressions of four genes (*CoWUS*, *CoBEL1a*, *CoBEL1b*, and *CoKAN4a*) and the similar transcriptome profiles between cluster 5 and 9 (Pearson’s correlation, r = 0.924) (**Supplementary Figure S5**). Four marker genes *CoSPLb*, *CoKAN4a*, *CoKAN4b*, and *CoSTK*, whose homologue (*AtSTK*) was expressed in the septum and ovule at anthesis (Herrera-Ubaldo et al., 2019), were detected to be highly expressed in cluster 2. Then, we suggested that both clusters 2 and 12 were from the inner integument (II) because of high expression of *CoSPLa* in cluster 12 and the similarity of transcriptome profiles between both clusters (Pearson’s correlation, r = 0.950) (**Supplementary Figure S5**).

Besides ovules, *AtKAN4* expression was visible in the vascular bundle of carpel (McAbee et al., 2006). *AT2G33860* is a marker gene of the carpel vascular system in PCMDB (Jin et al., 2022). *Solyc03g114840* was reported to be highly expressed in the pericarp of wild tomato at anthesis (Pattison et al., 2015). We found that *CoMADS2* (a homologue of *Solyc03g114840*), *CoKAN4a*, and *CoARF15* (a homologue of *AT2G33860*) were highly expressed in clusters 14–16, respectively (**Figure 3**; **Supplementary Table S10**). Therefore, we suggested that clusters 14–16 were from PIL, clusters 1 and 8 might also be from PIL, and cluster 6 might be from both the placenta and PIL.

### 3.5 Overviews of differentially expressed genes between CoXJ and CoGW

To explore the mechanisms of ovule abortion in *C. oleifera*, a comprehensive comparison of gene expression between CoXJ (a low-OAR variety) and CoGW (a high-OAR variety) was conducted. In total, 69,608 DE genes (**Supplementary Tables S11**-**S32**), whose potential functions and pathways were determined according to KEGG and GO analyses, were identified between CoXJ and CoGW in 18 clusters (**Supplementary Tables S12**-**S29**) and three combinations of clusters (**Supplementary Tables S30**-**S32**).

The proportion of downregulated genes was approximately 34.4%–91.7% in the 18 clusters and three combinations of clusters, and was approximately 48.5% on average (**Supplementary Figure S6**; **Supplementary Table S11**). The proportion of downregulated genes in clusters 2, 5, 9, 12, and 17 were approximately 41.3%, 51.3%, 50.2%, 44.0%, and 86.8%, respectively. The great difference in the proportions of downregulated genes between cluster 17 (female gametophyte cells) and clusters 2, 5, 9, and 12 (integument cells) was reflected in GO analyses (**Supplementary Figures S7A–E**). The proportion of downregulated genes in clusters 14–16 (VB cells) were approximately 74.0%, 79.4%, and 91.7%, respectively, which was also reflected in GO analyses (**Supplementary Figures S7F, G**). The abovementioned statistics data indicated that divergences of female gametophyte cells and VB cells were more than those of other cell types between CoXJ and CoGW.

In the 69,608 DE genes, 15 upregulated genes (**Supplementary Table S33**) and 70 downregulated genes (**Supplementary Table S34**) appeared in all 18 clusters. In the 70 downregulated genes, there were four aquaporins including three plasma membrane intrinsic proteins (CoPIP1-4, CoPIP2-4, and CoPIP2-5) and one tonoplast intrinsic protein (CoTIP1-3), four ethylene related proteins including S-adenosylmethionine synthase (CoSAM1) and three ethylene-responsive transcription factors (CoERF011, CoERF110a, and CoERF110b), and other transcription factors (CoBHLH162, CoBHLH35, CoKANT3, CoMYBS3, and CoWRKY6). Physiologically, the reduced CoPIP and CoTIP expression in CoGW could disrupt water influx to the ovary and ovule, contributing to ovule abortion. Six aquaporins (AtPIP1-5, AtPIP2-4, AtPIP2B, AtPIP2-3, AtTIP1-3, and AtTIP5-1) were downregulated in the *AtCWINV*-silenced transgenic plants, but their abortion ovules randomly distributed along the silique (Liao et al., 2020). Therefore, four aquaporins might be not directly related to ovule abortion in *C. oleifera*.

### 3.6 The overall expression of CoSWEET and CoCWINV upregulated in clusters 6 and 14 of CoXJ

The pattern of infertile ovules implied that there was selective abortion caused by resource limitation in *C. oleifera*. Some DE genes related to soluble sugar transport in ovaries, such as *CoSWEET1b* and *CoSWEET17d* in cluster 6 (**Supplementary Table S18**) and *CoSTP13a* in clusters 2, 5, 9, 12, and 17 (**Supplementary Tables S14**, **S17**, **S21**, **S24**, **S29**), were significantly upregulated in CoXJ. We identified genes from seven families (**Supplementary Figures S8A–F**; **Supplementary Table S8**) including SWEET (sugar will eventually be exported transporter), ACINV (acid invertase, including CWINV and INV [vacuole acid invertase]), ANInv (neutral/alkaline invertase), SUS (sucrose synthase), STP (sugar transporter protein), PLT (polyol transporter), and SUC (sucrose transporter) in *C. oleifera* based on phylogenetic analysis using MEGA7 (Kumar et al., 2016).

**Figure 4:**
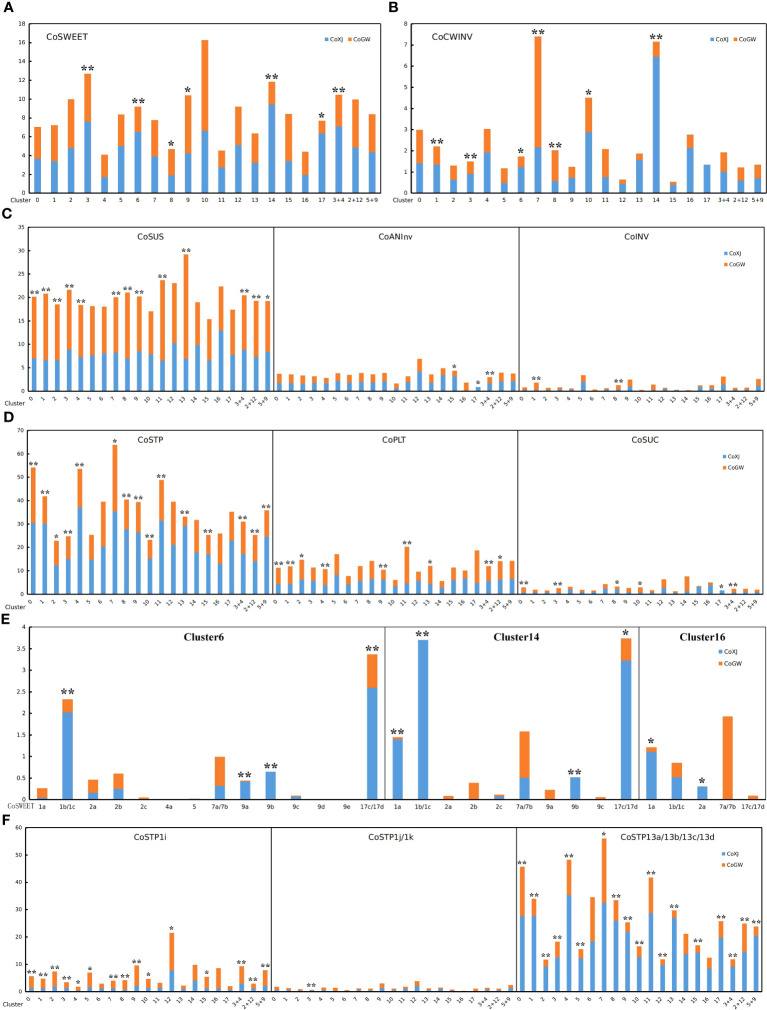
Expression values of the genes related to sugar transport in different clusters of CoXJ and CoGW. The analysis of variance was used for statistical analysis (**p< 0.01; *p< 0.05). **(A)** The overall expression of *CoSWEET*. **(B)** The overall expression of *CoCWINV*. **(C)** The overall expression of *CoSUS*, *CoANInv* and *CoINV*. **(D)** The overall expression of *CoSTP*, *CoPLT*, and *CoSUC*. **(E)** Expression of *CoSWEET* genes in clusters 6, 14, and 16. **(F)** Expression of *CoSTP1i*, *CoSTP1j/k*, and *CoSTP13a/13b/13c/13d*.

Differential expression analysis showed that the overall expression of *CoSWEET* and *CoCWINV* in both clusters 6 and 14 (**Supplementary Table S35**), which were procambium cells to transport sugar, was markedly higher in CoXJ than in CoGW (**Figures 4A, B**), while there were no significant differences in the overall expression of *CoINV*, *CoANInv*, *CoSUS*, *CoSTP*, *CoPLT*, and *CoSUC* (**Figures 4C, D**). The overall expression of *CoSWEET* in clusters 6 and 14 of CoXJ was 2.41- and 3.91-fold higher than those of CoGW, respectively. The overall expression of *CoCWINV* in clusters 6 and 14 of CoXJ was 2.47- and 8.87-fold higher than those of CoGW, respectively.

The overall expression of *CoSWEET* in CoXJ was 1.51-, 4.74-, and 2.10-fold higher in clusters 3 and 17, and in combination of clusters 3 and 4, and approximately 30% and 46% lower in clusters 8 and 9 than those of CoGW, respectively. The overall expression of *CoCWINV* in CoXJ was 1.56-, 1.57-, and 1.75-fold higher in clusters 1, 3, and 10, and approximately 142% and 163% lower in clusters 7 and 8 than those of CoGW, respectively. Both clusters 1 and 8 were PIL parenchyma cells. Thus, the overall expression of *CoCWINV* in PIL might be similar between CoXJ and CoGW.

A total of 21 *CoSWEET* genes (**Supplementary Figures S8A**, **S9**) were identified in *C. oleifera* genome by comparing with *CsSWEET*s in tea tree (Wang et al., 2018; Yao et al., 2020; Jiang et al., 2021). The expression of *CoSWEET1b/1c* (1b could not be distinguished from 1c), *CoSWEET17c/17d* (17c could not be distinguished from 17d), *CoSWEET9a*, and *CoSWEET9b* in cluster 6, and the expression of *CoSWEET1a*, *CoSWEET1b/1c*, *CoSWEET17c/17d*, and *CoSWEET 9b* in cluster 14, were highly upregulated in CoXJ compared with that in CoGW (**Figure 4E**; **Supplementary Table S36**). Seven *CoCWINV* genes were identified in *C. oleifera* (**Supplementary Figure S8B**). Although CoCWINV1c was one of the major cell wall acid invertases in ovaries, no significant difference was detected on its expression in clusters 6 and 14 between CoXJ and CoGW (**Supplementary Figure S10**). Compared with CoGW, a higher expression of *CoCWINV2a* and *CoCWINV2b* in cluster 6, and a higher expression of *CoCWINV1b*, *CoCWINV2a*, *CoCWINV2b*, *CoCWINV2c*, and *CoCWINV2d* in cluster 14,= resulted in higher overall expression of *CoCWINV* in CoXJ.

Five *CoINV* genes were identified in *C. oleifera* (**Supplementary Figure S8B**). CoINV2a and CoINV2b were major vacuole acid invertases, but their expression was very low in clusters 6 and 14 (**Supplementary Figure S11**). A total of 10 *CoANInv* genes were identified in *C. oleifera* (**Supplementary Figure S8D**). Major neutral/alkaline invertases were CoANInvB1, CoANInvB2, CoANInvE2, and CoANInvI1. The expression of *CoANInvB1* and *CoANInvB2* was highly upregulated in cluster 14 of CoXJ, while no significant difference was found on their expression in cluster 6 between CoXJ and CoGW (**Supplementary Figure S12**). Five *CoSUS* genes were identified in *C. oleifera* (**Supplementary Figure S8C**). CoSUS3 and CoSUS4 were major sucrose synthases and were highly upregulated in most ovary cells except for most VB cells and part ovule cells of CoGW (**Supplementary Figure S13**).

### 3.7 Subcellular localization and substrat prediction of CoSWEETs

**Figure 5:**
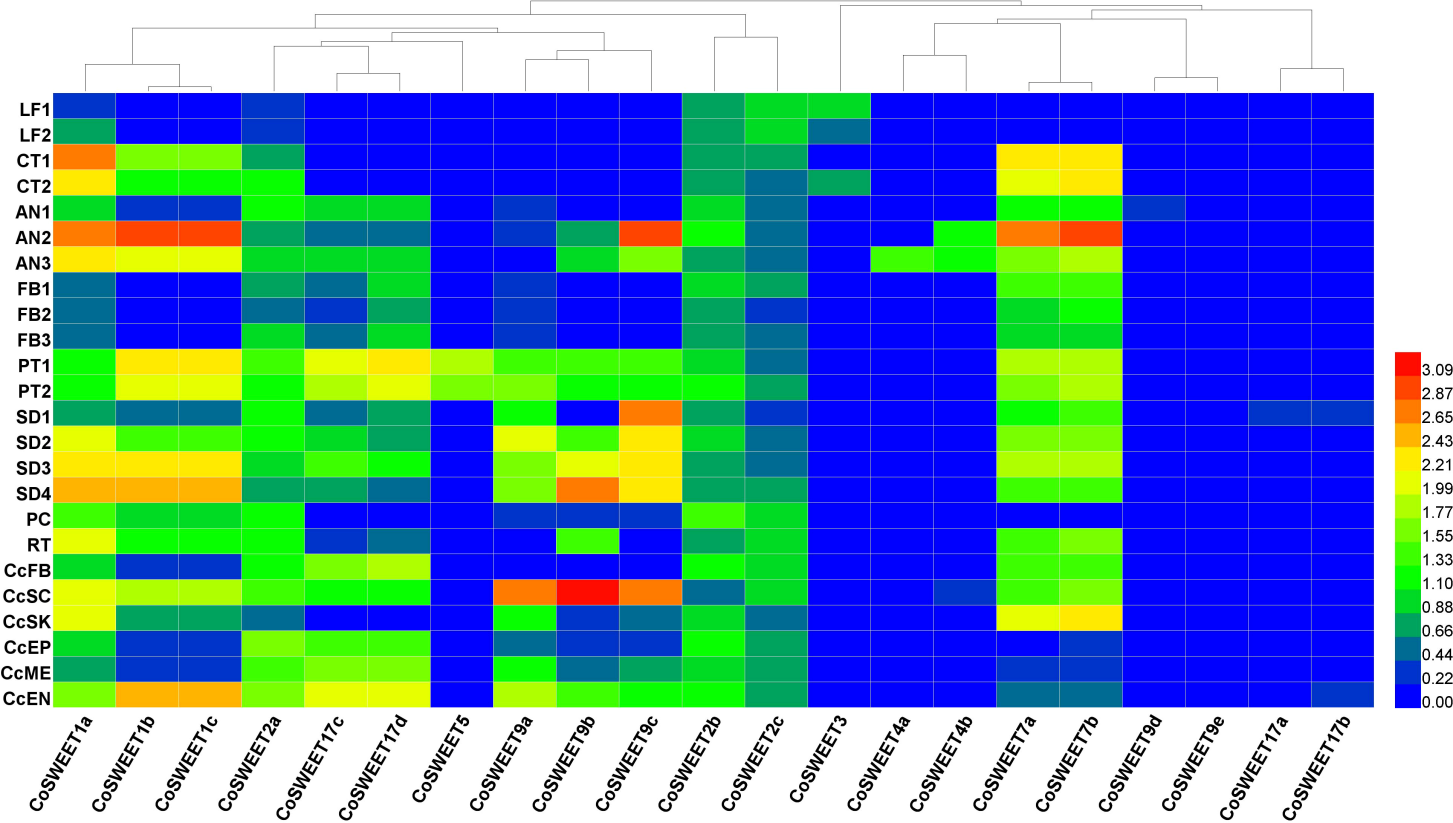
Heat map of *CoSWEET* expression in different tissues. Different colors indicated different levels of gene expression: from red to blue, the log_10_ (FPKM + 1) value ranged from large to small. LF1, annual leaves in August, CRR180020-22; LF2, biennial leaves in August, CRR180023-25; CT1, cotyledon tissue from seeds broken in shell, SRR17155283-85; CT2, cotyledon tissue from seedlings with 10 cm root, SRR17155290-92; AN1, anthers in pollen mother cell stage, CRR400359-61; AN2, anthers in tetrad stage, CRR400362-64; AN3, anthers in uninucleate pollen stage, CRR400365-67; FB1, FB (flower buds without sepals and pedicels) in June, CRR274890-92; FB2, FB in July, CRR274896-98; FB3, FB in August, CRR274905-07; PT1, self-pollinated pistils after 48–75 h, SRR8275897-99; PT2, cross-pollinated pistils after 48–75 h, SRR8275900-02; SD1, seeds in July, SRR13493696-98; SD2, seeds in August, SRR13493693-95; SD3, seeds in September, SRR13493690-92; SD4, seeds in October, SRR13493686-88; PC, pericarp in November, SRR21160439-41; RT, root, SRR23501938-40; CcFB, Cc (*Camellia chekiangoleosa*) flower buds, SRR10121546/48/49; CcSC, Cc seed coat, SRR10121540-42; CcSK, Cc seed kernel, SRR10121556/57/59; CcEP, Cc epicarp, SRR10121550-52; CcME, Cc mesocarp, SRR10121543-45; CcEN, Cc endocarp, SRR10121553-55.

The functional implications of *CoSWEET* genes could be addressed through subcellular localization and expression analysis. We collected transcriptome data from different tissues, including leaf, cotyledon, root, flower bud, anther, pistil, seed, seed coat, seed kernel, pericarp, epicarp, mesocarp, and endocarp (**Figure 5**). The CoSWEETs were predicted using WoLF PSORT (http://wolfpsort.org) (Horton et al., 2007) to localize to the plasma or vacuolar membranes in general (**Supplementary Table S37**), but their localizations to the membranes of organelles such as the endoplasmic reticulum, Golgi apparatus, or chloroplast could not be excluded.

CsSWEET1a (a homologue of CoSWEET1a) was a plasma membrane-localized glucose transporter, and CsSWEET17 (a homologue of CoSWEET17c/17d) could transport both glucose and fructose across plasma membrane (Yao et al., 2020). Only *CoSWEET1a*, *CoSWEET1b*/*1c*, and *CoSWEET2a* were detected in cluster 16 (**Figure 4E**). Therefore, we speculated that hexose and sucrose efflux across plasma membrane in cluster 16 might be through CoSWEET1a and CoSWEET1b/1c because CoSWEET2a might be localized to the vacuole membrane and that CoSWEET17c/17d might be the same as CsSWEET17 in subcellular localization and substrat. The expression of *CoSWEET9a*, *CoSWEET9b*, and *CoSWEET9c* was highly upregulated in the seed coat (**Figure 5**), which was similar with *AtSWEET11*, *AtSWEET12*, and *AtSWEET15* (Chen et al., 2015). Therefore, we speculated that CoSWEET9a, CoSWEET9b, and CoSWEET9c were plasma membrane-localized sucrose transporters. *CoSWEET7a/7b* (7a could not be distinguished from 7b) was highly expressed in anther, cotyledon, and seed. There was no significant difference in the expression of *CoSWEET7a/7b* in clusters 6 and 14 between CoXJ and CoGW (**Figure 4E**). CoSWEET7a/7b might be similar with VvSWEET7 (Breia et al., 2020) and SlSWEET7 (Zhang et al., 2021), which were plasma membrane-localized sucrose/hexose transporters.

CsSWEET3 (a homologue of CoSWEET3) was downregulated by cold acclimation (Yue et al., 2015) and mannitol-induced osmotic stress (Samarina et al., 2020) and upregulated by biotrophic pathogen *Colletorichum camelliae* infection (Yao, 2020). CoSWEET3, which was preferentially expressed in the leaf and cotyledon, might be the same as ClSWEET3, which is a plasma membrane-localized hexose transporter (Ren et al., 2021). CoSWEET4 was highly expressed in anthers and might be similar with AtSWEET4, which is a plasma membrane-localized hexose transporter (Liu et al., 2016). CoSWEET17a/17b (17a could not be distinguished from 17b) might be the same as CsSWEET16, which was a vacuolar membrane-localized hexose transporter (Wang et al., 2018). It is interesting that CoSWEET5, whose homologue AtSWEET5 was a plasma membrane-localized hexose transporter highly expressed in mature pollen grains (Wang et al., 2022b), was only expressed in cross- and self-pollinated pistils.

### 3.8 CoSTP13 genes upregulated in ovule cells of CoXJ

Among proton-dependent transporters (including CoSTP, CoPLT, and CoSUC) connected to sugar uptake from the apoplast, the overall expression of *CoSTP* was the highest, and *CoSUC* was the lowest. The overall expression of *CoSTP* in the integument was significantly upregulated in CoXJ. The overall expression of *CoSTP* in cluster 9 of CoXJ was 2.02-fold higher than that in CoGW, 1.18-fold higher in cluster 2, 2.19-fold higher in combination of clusters 5 and 9, and 1.25-fold higher in combination of clusters 2 and 12 (**Figure 4D**).

A total of 30 *CoSTP* genes were identified in *C. oleifera* genome (**Supplementary Figure S8E**). In ovaries, STPs mainly were CoSTP1i, CoSTP1j/k (1j could not be distinguished from 1k), and CoSTP13a/13b/13c/13d (13a, 13b, 13c, and 13d could not be distinguished from each other) (**Supplementary Figure S14**). The expression of *CoSTP13a/13b/13c/13d* in clusters 2, 5, 9, 12, and 17 of CoXJ was 3.38-, 3.46-, 6.21-, 4.67-, and 3.23-fold higher than that in CoGW, respectively, and 3.51-, 1.41-, and 5.81-fold higher in combination of clusters 3 and 4, in combination of clusters 2 and 12, and in combination of clusters 5 and 9, respectively (**Supplementary Table S36**). In contrast to *CoSTP13a/13b/13c/13d*, the expression of *CoSTP1i* in clusters 2, 5, 9, and 12 of CoGW was significantly higher than that in CoXJ. However, the expression of *CoSTP1i* was lower than *CoSTP13a/13b/13c/13d*. The expression of *CoSTP1j/k* was the lowest (**Figure 4F**).

A total of 16 *CoPLT* genes were identified in *C. oleifera* (**Supplementary Figure S8E**). Although the overall expression of *CoPLT* was approximately 49% higher in cluster 9 of CoXJ than that of CoGW, it was approximately 32% lower in cluster 2. The overall expression of *CoPLT* had no significant difference in clusters 5, 12, and 17 between CoXJ and CoGW (**Figure 4D**). In ovaries, major PLTs were CoPLT2c, CoPLT2d, CoPLT2e, CoPLT2f, CoPLT2g, and CoPLT2i (**Supplementary Figure S15**). A total of 15 *CoSUC* genes were identified in *C. oleifera* (**Supplementary Figure S8F**). Except for cluster 17, in which no *CoSUC* was detected in CoGW, the overall expression of *CoSUC* had no significant difference in the ovary cells between CoXJ and CoGW. In ovaries, major SUCs were CoSUC2a, CoSUC2d, CoSUC2e, CoSUC2f, CoSUC2g, CoSUC2h, CoSUC2i, and CoSUC4, which might be the same as AtSUC4 that was localized to vacuole membrane (Endler et al., 2006). The expression of *CoSUC3a* and *CoSUC3b* was very low (**Supplementary Figure S16**).

## 4 Discussion

### 4.1 CoXJ was a superior low-OAR variety

Since the phenomenon of ovule abortion in *C. oleifera* was reported by Cao (1965), investigations on mechanisms of ovule abortion have been done by different researchers (Zhou et al., 1991; Chen et al., 2014; Liao et al., 2014; Gao, 2017) who usually utilized middle- or high-OAR varieties. CoXJ was a newly discovered low-OAR variety with an OAR of 28.9% on average in open pollination and even had no abortion ovule in some fruits bearing upper outside the canopy. The identification of CoXJ opened a new door to comparatively study normal ovule development and ovule abortion in *C. oleifera*. In addition, CoXJ was an elite germplasm resource for high-yield breeding.

### 4.2 Construction of a single-cell atlas of *C. oleifera* mature ovaries

The ovary was a highly heterogeneous tissue including the ovule, placenta, and pericarp. In this study, we captured the major cell types of *C. oleifera* mature ovaries to construct a high-resolution transcriptome atlas. This knowledge will facilitate future work on ovary development at single-cell resolution and serve as a resource to understand cell fate determination during the ovary maturing process.

Cell definition is the key step and greatest challenge to overcome in the application of single-cell sequencing in non-model plants. Because there was no exact marker gene for each *C. oleifera* ovary cell type, we employed multiple known orthologous marker genes from tea tree and *Arabidopsis* to annotate *C. oleifera* ovary cell types. After identification of FG and VB cells, we employed another strategy that had been used in the corolla of wild tobacco (Kang et al., 2022) to define EP cells, in which DE genes involved in cutin and cuticular wax biosynthesis were enriched, and to define PH cells, in which DE genes involved in photosynthesis were enriched. Because there were no marker genes to determine their identities, we could not determine the cell types of clusters 0, 5, 8, and 11, which were defined as putative PH cells without photosynthesis.

Multiple known tissue-specific orthologous genes from *Arabidopsis* and wild tomato were used to annotate *C. oleifera* ovary tissue types. Definition of cell types also provided clues to identify tissue types of cell clusters. However, we could not determine the tissue types of clusters 1, 6, and 8 owing to lack of tissue-specific genes to determine their identities. We speculated that clusters 1 and 8 might be from PIL because there were many PH cells with or without photosynthesis in PIL, and cluster 6 might be from both PL and PIL because its cell number was the largest in VB cells (**Supplementary Table S5**), and we had defined clusters 14–16 as VB cells from PIL, while there existed VB cells in PL (Gao, 2017).

### 4.3 Identification of the genes related to ovule abortion in *C. oleifera*

We identified 69,608 DE genes between CoXJ and CoGW. Two clues were obtained by statistically analyzing the DE genes. One was that divergences of female gametophyte cells and VB cells were more than those of other cell types between CoXJ and CoGW. Another was that four aquaporins (CoPIP1-4, CoPIP2-4, CoPIP2-5, and CoTIP1-3) were downregulated in CoGW and could disrupt water influx to the ovary and ovule, contributing to ovule abortion. In the *AtCWINV*-silenced transgenic plants, six aquaporins were downregulated (Liao et al., 2020). The reduced aquaporins might be caused by low soluble sugar in CoGW and the *AtCWINV*-silenced transgenic plants.

According to the phenotype that infertile ovules were located in the middle and lower sections of the ovary (Gao, 2017), we suggested that the infertile ovules was caused by selective abortion in *C. oleifera* owing to assimilate limitation. Single-cell RNA-seq gave us an advantage that we could know cell types and their principal functions. Therefore, the expression of genes related to sugar transport in different clusters, especially clusters 6, 14, and 16, which were VB cells to transport sugar, was checked. The result showed that the overall expressions of *CoSWEET* and *CoCWINV* in both clusters 6 and 14 of CoXJ was markedly higher than those of CoGW. Finally, we suggested that *CoSWEET1b/1c*, *CoSWEET9a*, *CoSWEET9b*, *CoSWEET17c/17d*, *CoCWINV2a*, *CoCWINV2b*, and *CoSTP13a/13b/13c/13d* might be related to ovule abortion in *C. oleifera*.

### 4.4 Sugar unloading from procambium cells to the apoplast

**Figure 6:**
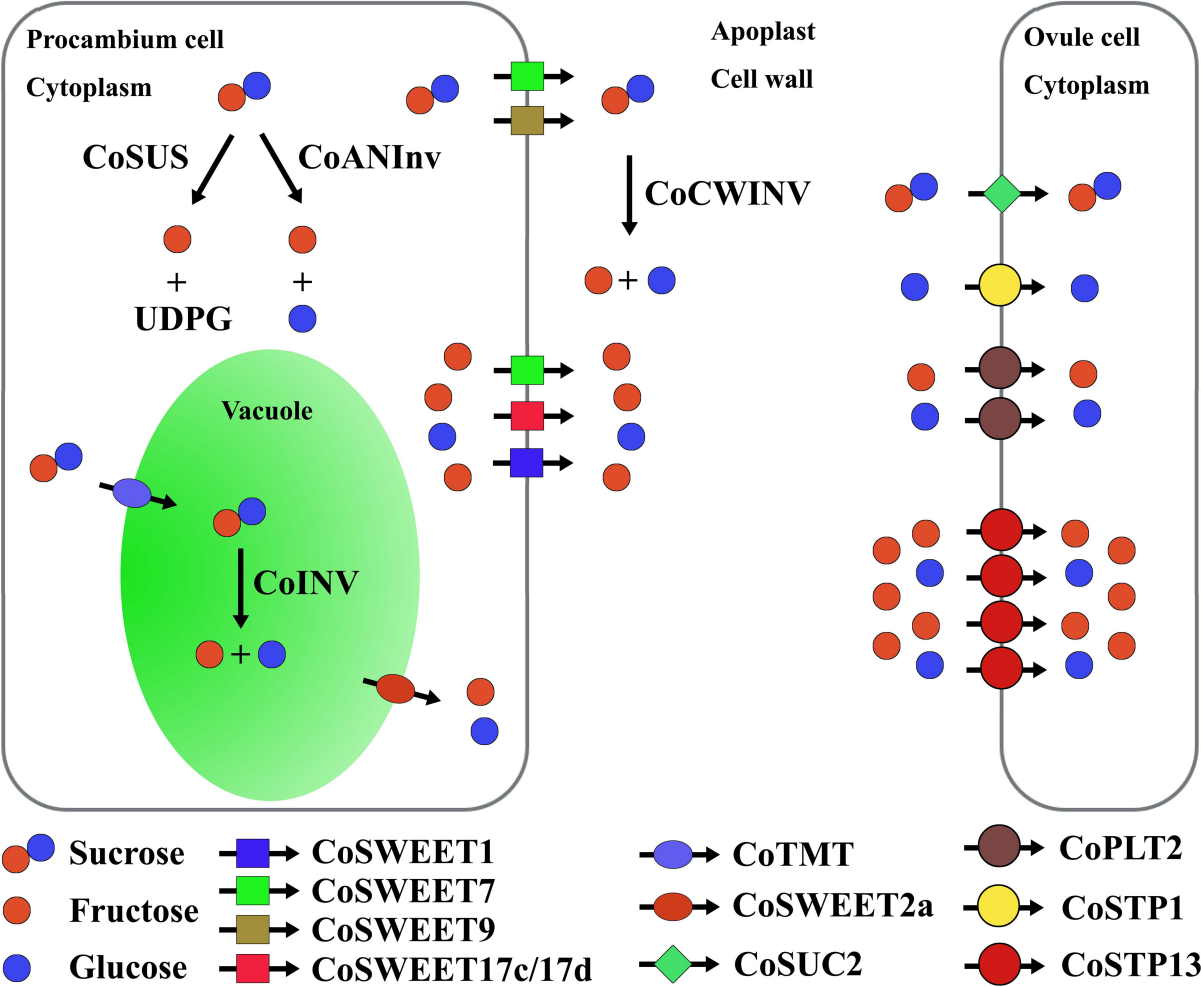
Sugar unloading from procambium cells to the apoplast and uptake from the apoplast into ovule cells. Co, *Camellia oleifera*; SWEET, sugar will eventually be exported transporter; SUS, sucrose synthase; ANInv, neutral/alkaline invertase; INV, vacuole acid invertase; CWINV, cell wall acid invertase; TMT, tonoplast monosaccharide transporter; SUC, sucrose transporter; PLT, polyol transporter; STP, sugar transporter protein.

Based on predicted subcellular localization and substrats of CoSWEETs and the expression of genes connected to the sucrose cleavage in the cytosol, we suggested that the sugar unloading from procambium cells to the apoplast was generally through CoSWEET1, CoSWEET7, CoSWEET9, and CoSWEET17c/17d, and that the major sugar were sucrose and hexose, and the major hexose was fructose (**Figure 6**). In detail, sucrose efflux might be through CoSWEET7 and CoSWEET9, while hexose efflux might be through CoSWEET1, CoSWEET7 and CoSWEET17c/17d.

Among genes (including *CoSUS*, *CoANInv*, and *CoINV*) connected to the sucrose cleavage inside the cell, the overall expression of *CoSUS* was the highest, while *CoINV* was the lowest (**Figure 4C**). CoINV might contribute little to the sucrose cleavage in sugar transport. Therefore, the major hexose in the cytosol might be fructose, which was largely from the CoSUS-catalyzed reversible conversion of sucrose and partly from sucrose cleavage catalyzed by CoANInv.

The upregulation of *CoSWEET* overall expression in procambium cells of CoXJ was caused by the higher expression of *CoSWEET1b/1c*, *CoSWEET17c/17d*, *CoSWEET9a*, and *CoSWEET9b* in cluster 6 and the higher expression of *CoSWEET1a*, *CoSWEET1b/1c*, *CoSWEET17c/17d*, and *CoSWEET 9b* in cluster 14. Although the overall expression of *CoSUS* was similar in procambium cells of both CoXJ and CoGW, more fructose in CoXJ might be created by CoSUS-catalyzed sucrose cleavage and exported to the apoplast owing to the upregulation of passive diffusive hexose transporters such as CoSWEET17c/17d (3.32- and 6.22-fold increasement in clusters 6 and 14 of CoXJ, respectively).

CoXJ might have a significantly high efflux activity to sucrose and hexose by incorporating different CoSWEETs. The upregulation of *CoCWINV* overall expression would enhance CWINV activity in the cell wall and further promote sucrose efflux from procambium cells to the apoplasm by hydrolyzing sucrose into glucose and fructose in CoXJ.

### 4.5 Sugar uptake from the apoplast into ovule cells

Sugar uptake from the apoplast is essential for symplastically isolated cells like pollen, guard cells, cells of the inner integument of the seed coat, the endosperm and the embryo, and egg cells (Rottmann et al., 2018). It is unknown whether cells of the inner integument is symplastically isolated from the outer integument in *C. oleifera* mature ovaries. However, the unloading pathway of soluble sugars from sieve element–companion cell complex might be predominantly apoplasmic in cucumber fruit from anthesis to the marketable maturing stage (Hu et al., 2011). In *Arabidopsis*, phloem-mobile fluorescent tracers were unloaded into cells of ovule primordia following a symplastic pathway and could not move out of phloem cells into mature ovules (Werner et al., 2011).

AtSTP1 (a homologue of CoSTP1) protein is a high-affinity monosaccharide/H^+^ symporter localized to plasma membrane and is able to transport a suite of hexoses, but not fructose (Sauer et al., 1990; Stadler et al., 2003). The plasma membrane-localized AtSTP13 (a homologue of CoSTP13) protein can transport both glucose and fructose (Norholm et al., 2006; Liu et al., 2021b). AtPMT2 (AT2G16130, a homologue of CoPLT2) protein is a glucose, fructose, and xylitol/H^+^ symporter localized to the plasma membrane in pollen and young xylem cells (Klepek et al., 2010). AtSUC2 (a homologue of CoSUC2) is a membrane-localized sucrose/H^+^ symporter (Sauer and Stolz, 1994). SWEET proteins usually have higher K_m_ values for sugar uptake than efflux; for example, AtSWEET12 (a homologue of CoSWEET9b) has K_m_ values of 70 mM and 10 mM for sucrose uptake and efflux, respectively (Chen et al., 2012), while AtSUC2 has a K_m_ of 0.77 mM for sucrose uptake (Sauer and Stolz, 1994). Therefore, the sugar uptake from the apoplast in *C. oleifera* mature ovules might be through CoSTP1, CoSTP13, CoPLT2, and CoSUC2 rather than CoSWEET proteins, which might take part in sugar efflux.

In *C. oleifera* mature ovules, most sucrose in the apoplast might be converted to hexose by CoCWINV, and then, the hexose in the apoplast was absorbed through monosaccharide transporters CoSTP1, CoSTP13, and CoPLT2 because the overall expression of *CoSUC* was the least among *CoSTP*, *CoPLT*, and *CoSUC.* Glucose uptake might be mainly through CoSTP1, CoSTP13, and CoPLT2, while fructose uptake might be mainly through CoSTP13 and CoPLT2.

AtSTP1 has a K_m_ of approximately 20 μM for glucose (Sauer et al., 1990). AtSTP13 mediates glucose uptake that follows saturation kinetics with an apparent K_m_ value of 74 ± 14 μM (Norholm et al., 2006). AtPMT2 has a K_m_ of approximately 0.18 mM for xylitol and a K_m_ of approximately 1.25 mM for glucose (Klepek et al., 2010). A higher K_m_ value of AtPMT2 for hexose implied that fructose uptake from the apoplast might rely on CoSTP13 rather than CoPLT2 in *C. oleifera* mature ovules. The overall expression of *CoSTP* was the highest among *CoSTP*, *CoPLT*, and *CoSUC*. Therefore, the monosaccharide transporters, especially CoSTP13, might play a central role in sugar absorption from the apoplast. The remarkedly upregulating expression of *CoSTP13* in CoXJ not only increased overall expression of *CoSTP* but also promoted fructose uptake from the apoplast and sugar unloading in procambium cells, in which exported hexoses might mainly be fructose.

### 4.6 Possible mechanism of ovule abortion in *C. oleifera*

Except for self-incompatibility, mechanisms of ovule abortion lasting from the stage of embryonic sac maturity to the stage of early zygote in *C. oleifera* might be selective abortion caused by low sugar levels in the apoplast around procambium cells and a low capability of hexose uptake in the integument. Selective abortion might be the main mechanism of ovule abortion in *C. oleifera*. Among the linearly arranged ovules, ovules on the apical region of the ovary might be the nearest to procambium cells and obtain enough assimilates, while the ones on the basal end of the ovary might be the farthest from maternal resources and abort preferentially.

In *Arabidopsis*, silencing *AtCWINV2* and *AtCWINV4* inhibited ovule initiation and induced ovule abortion, and genes of hexose transporter such as AtSWEET3, AtSWEET4, AtSWEET5, AtSWEET7, AtSWEET8, AtSTP2, AtSTP6, and AtSTP9 were all downregulated in the *AtCWINV*-silenced transgenic plants (Liao et al., 2020). The expression of *CoSWEET1b/1c*, *CoSWEET9a*, *CoSWEET9b*, *CoSWEET17c/17d*, *CoCWINV2a*, *CoCWINV2b*, and *CoSTP13a/13b/13c/13d* was coordinated upregulated, and ovules on the basal end of the ovary might absorb enough assimilates and develop normally in mature ovaries of low-OAR variety, while ovules on the basal end of the ovary might abort owing to lack of assimilates in mature ovaries of high-OAR variety. The reduced CoPIP and CoTIP expression in CoGW might contribute to ovule abortion. However, it was unknown how dynamics in sugar metabolism signaling might regulate aquaporin gene expression (Liao et al., 2020). The pattern of infertile ovules, normal transmitting tract (Liao, 2013; Gao, 2017; Gao et al., 2019), and our research results have provided strong experimental evidence for selective abortion in *C. oleifera.* However, the transcriptome atlas of *C. oleifera* mature ovaries needs to be improved, the subcellular localization and substrats of CoSWEETs should be confirmed by experiments, and the differentiated structure and function of the vascular tissue in pericarp and placenta need further studies. We will integrate spatial transcriptomics and single-cell RNA-seq to research development of vascular bundle and sugar unloading in pericarp and placenta next. The regulation of sugar transport in ovaries might be a new horizon to overcome selective abortion and enhance seed yield in *C. oleifera.*

## 5 Conclusion

Overall, we constructed the first single-cell transcriptional landscape in woody crop ovaries. The overall expression of *CoSWEET* and *CoCWINV* in procambium cells and *CoSTP* in the integument was significantly upregulated in low-OAR variety CoXJ owing to the upregulation of genes such as *CoSWEET1b/1c*, *CoSWEET9a*, *CoSWEET9b*, *CoSWEET17c/17d*, *CoCWINV2a*, *CoCWINV2b*, and *CoSTP13a/13b/13c/13d*. Our investigation reveals a link of ovule abortion and sugar transport and sheds light on further deciphering the mechanism of regulating sugar transport and the improvement of seed yield in *C. oleifera*.

## Data availability statement

The original contributions presented in the study are included in the article/**Supplementary Material**, further inquiries can be directed to the corresponding author.

## Author contributions

SZ: Conceptualization, Data curation, Formal analysis, Funding acquisition, Investigation, Methodology, Writing – original draft, Writing – review & editing. JR: Conceptualization, Methodology, Writing – original draft, Writing – review & editing.
