# Supplementary material for: Single-cell RNA-seq reveals a link of ovule abortion and sugar transport in Camellia oleifera
Source: Front Plant Sci. 2024 Feb 2;15:1274013. doi: 10.3389/fpls.2024.1274013 (PMC10869455; doi:10.3389/fpls.2024.1274013)
Supplement: Supplementary file 1 [file DataSheet_1.zip › Supplementary Figures 1-16.doc]

**Figure S1.** Opened fruits of *Camellia oleifera*. **(A)** CoXJ, *C. oleifera* variety ‘XJ’. **(B)** CoGW, *C. oleifera* variety ‘GW’. The arrows indicated aborted seeds.

**A	C**

**B	D**

**Figure S2.** Quality control of scRNA-seq. **(A, B)** Cells with gene (Feature_RNA) numbers from 360 to 4,000 were retained. The cells with fewer than 360 genes were considered low quality, and the cells with more than 4,000 genes were likely to be two or more cells in one drop. **(C, D)** Cells with 22,000 UMIs (Count_RNAs) were retained. The cells with UMI numbers more than 22,000 were likely two or more cells in one drop. **(A, C)** Before filter; **(B, D)** after filter. CoGW: *Camellia oleifera* variety ‘GW’. CoXJ: *C. oleifera* variety ‘XJ’.

**Figure S3.** Hierarchical clustering of 31 cells in cluster 17. Analysis of hierarchical cluster was based on the expressing values of top 1000 variable genes in cluster 17. EC, egg cell; SC, synergid cell; GW, *Camellia oleifera* cultivar ‘GW’; XJ, *C. oleifera* cultivar ‘XJ’; C17, cluster 17.

| **A** Cluster 1 | **B** Cluster 3 |
| --- | --- |
|  |  |
|  |  |
| **C** Cluster 4 | **D** Cluster 10 |
|  |  |
|  | |
| **E** Cluster 2 | |
|  | |

**Figure S4.** GO or KEGG enrichment analysis of the DE genes in five cell clusters. **(A)** The cellular components of ‘photosystem II’ (GO:0009523) were enriched in cluster 1. **(B)** The cellular components of ‘photosystem’ (GO:0009521), ‘photosynthetic membrane’ (GO:0034357) and ‘photosystem I’ (GO:0009522) were enriched in cluster 3. **(C)** The cellular components of ‘photosystem I’ were enriched in cluster 4. **(D)** The cellular components of ‘photosystem’, ‘photosynthetic membrane’, ‘photosystem I’ and ‘photosystem II’ were enriched in cluster 10. **(E)** KEGG pathway ‘photosynthesis’ (ko00195) was enriched in cluster 2. Clusters 1, 2, 3, 4 and 10 were defined as parenchyma cells.

**Figure S5.** Heat map of Pearson’s correlation coefficient of gene expression in different cell clusters.

**Figure S6.** Distribution of DE genes between CoXJ and CoGW. CoGW: *Camellia oleifera* variety ‘GW’. CoXJ: *C. oleifera* variety ‘XJ’.

| **A** Cluster 2 | **B** Cluster 5 |
| --- | --- |
|  |  |
|  |  |
| **C** Cluster 9 | **D** Cluster 12 |
|  |  |
|  |  |
| **E** Cluster 17 | **F** Cluster 14 |
|  |  |
|  |  |
| **G** Cluster 15 | **H** Cluster 16 |
|  |  |

**Figure S7.** GO enrichment analysis of the DE genes between CoXJ and CoGW in eight cell clusters. CoGW: *Camellia oleifera* variety ‘GW’. CoXJ: *C. oleifera* variety ‘XJ’.

**Figure S8.** Identification of the genes related to sugar transport based on phylogenetic analysis in *Camellia oleifera*. Neighbor-joining phylogenetic trees of SWEET **(A)**, ACINV **(B)**, SUS **(C)**, ANInv **(D)**, STP\PLT **(E)** and SUC **(F)** proteins were constructed using MEGA7, respectively.

**Figure S9.** Heat map of CoSWEET expression. The heat map showed expression values of 21 CoSWEET genes in 18 clusters and 3 combinations of the clusters. Different colors indicated different values of gene expression: from red to blue, the expression value ranged from large to small. CoXJ: *Camellia oleifera* variety ‘XJ’. CoGW: *C. oleifera* variety ‘GW’.

**Figure S10.** Heat map of *CoCWINV* expression. The heat map showed expression values of 7 *CoCWINV* genes in 18 clusters and 3 combinations of the clusters. Different colors indicated different values of gene expression: from red to blue, the expression value ranged from large to small. CoXJ: *Camellia oleifera* variety ‘XJ’. CoGW: *C. oleifera* variety ‘GW’.

**Figure S11.** Heat map of *CoVIN* expression. The heat map showed expression values of 5 *CoVIN* genes in 18 clusters and 3 combinations of the clusters. Different colors indicated different values of gene expression: from red to blue, the expression value ranged from large to small. CoXJ: *Camellia oleifera* variety ‘XJ’. CoGW: *C. oleifera* variety ‘GW’.

**Figure S12.** Heat map of *Co**ANInv* expression. The heat map showed expression values of 10 *CoANInv* genes in 18 clusters and 3 combinations of the clusters. Different colors indicated different values of gene expression: from red to blue, the expression value ranged from large to small. CoXJ: *Camellia oleifera* variety ‘XJ’. CoGW: *C. oleifera* variety ‘GW’.

**Figure S13.** Heat map of *CoSUS* expression. The heat map showed expression values of 5 *CoSUS* genes in 18 clusters and 3 combinations of the clusters. Different colors indicated different values of gene expression: from red to blue, the expression value ranged from large to small. CoXJ: *Camellia oleifera* variety ‘XJ’. CoGW: *C. oleifera* variety ‘GW’.

**Figure S14.** Heat map of *CoSTP* expression. The heat map showed expression values of 30 *CoSTP* genes in 18 clusters and 3 combinations of the clusters. Different colors indicated different values of gene expression: from red to blue, the expression value ranged from large to small. CoXJ: *Camellia oleifera* variety ‘XJ’. CoGW: *C. oleifera* variety ‘GW’.

**Figure S15.** Heat map of *CoPLT* expression. The heat map showed expression values of 16 *CoPLT* genes in 18 clusters and 3 combinations of the clusters. Different colors indicated different values of gene expression: from red to blue, the expression value ranged from large to small. CoXJ: *Camellia oleifera* variety ‘XJ’. CoGW: *C. oleifera* variety ‘GW’.

**Figure S16.** Heat map of *CoSUC* expression. The heat map showed expression values of 15 *CoSUC* genes in 18 clusters and 3 combinations of the clusters. Different colors indicated different values of gene expression: from red to blue, the expression value ranged from large to small. CoXJ: *Camellia oleifera* variety ‘XJ’. CoGW: *C. oleifera* variety ‘GW’.
