# Supplementary material for: Single-cell RNA-seq reveals a link of ovule abortion and sugar transport in Camellia oleifera
Source: Front Plant Sci. 2024 Feb 2;15:1274013. doi: 10.3389/fpls.2024.1274013 (PMC10869455; doi:10.3389/fpls.2024.1274013)
Supplement: Supplementary file 1 [file DataSheet_1.zip › Supplementary Table 2.doc]

**Table S2.** Fruit and seed setting status of different cross combinations in *Camellia oleifera*.

| **Year** | **Cross combination** | | **Number of pollinated flowers** | **Number of harvested fruits** | **Fruit setting rate /%** | **Number of harvested seeds** | **Seed number per fruit** |
| --- | --- | --- | --- | --- | --- | --- | --- |
|  | **Female parent (variety)** | **Male parent (variety)** |  |  |  |  |  |
| 2011 | CoXJ | CoGW | 30 | 10 | 33.3 | 126 | 12.60 |
| 2011 | CoXJ | GW2 | 25 | 7 | 28.0 | 98 | 14.00 |
| 2017 | CoXJ | CoGW | 30 | 7 | 23.3 | 86 | 12.29 |
| 2018 | CoXJ | GW2 | 30 | 9 | 30.0 | 106 | 11.78 |
| 2021 | CoXJ | - |  | 65 |  | 755 | 11.62 |
| 2011 | CoGW | CoXJ | 245 | 106 | 43.3 | 316 | 2.98 |
| 2012 | CoGW | SJZ | 25 | 11 | 44.0 | 34 | 3.09 |
| 2015 | CoGW | - | 51 | 19 | 37.3 | 61 | 3.21 |
| 2016 | CoGW | - | 60 | 17 | 28.3 | 57 | 3.35 |
| 2021 | CoGW | - |  | 300 |  | 1015 | 3.38 |
| 2014 | GX46 | SJZ | 200 | 46 | 23.0 | 115 | 2.50 |
| 2014 | GX46 | SMTH | 500 | 109 | 21.8 | 265 | 2.43 |
| 2014 | GX46 | CoXJ | 100 | 20 | 20.0 | 24 | 1.20 |
| 2011 | GW2 | CoXJ | 197 | 149 | 75.6 | 715 | 4.80 |
| 2014 | GW2 | CM | 250 | 42 | 16.8 | 181 | 4.31 |
| 2014 | GW2 | - | 43 | 15 | 34.9 | 74 | 4.93 |
| 2015 | GW2 | - | 64 | 19 | 29.7 | 101 | 5.32 |
| 2016 | GW2 | - | 54 | 17 | 31.5 | 88 | 5.18 |
| 2012 | GW16 | CoGW | 25 | 13 | 52.0 | 28 | 2.15 |
| 2012 | GW16 | GW2 | 25 | 10 | 40.0 | 29 | 2.90 |
| 2012 | G8 | SMTH | 25 | 8 | 32.0 | 81 | 10.13 |
| 2013 | G8 | SJZ | 20 | 7 | 35.0 | 66 | 9.43 |
| 2014 | G8 | CoXJ | 300 | 76 | 25.3 | 450 | 9.87 |

- : open-pollination
